# A Study of the Effect of Sintering Conditions of Mg_0.95_Ni_0.05_Ti_3_ on Its Physical and Dielectric Properties

**DOI:** 10.3390/molecules25245988

**Published:** 2020-12-17

**Authors:** Chun-Hsu Shen, Chung-Long Pan, Shih-Hung Lin

**Affiliations:** 1 Department of Electronic Engineering, National Yunlin University of Science and Technology, Yunlin 64002, Taiwan; jameschs@yuntech.edu.tw; 2 Department of Electrical Engineering, I-Shou University, Kaohsiung 84001, Taiwan; ptl@isu.edu.tw

**Keywords:** microwave performances, sintering temperature, low-loss ceramics, Mg_0.95_Ni_0.05_TiO_3_

## Abstract

Mg_0.95_Ni_0.05_TiO_3_ ceramics were prepared by traditional solid-state route using sintering temperatures between 1300 and 1425 °C and holding time of 2–8 h. The sintered samples were characterized for their phase composition, micro-crystalline structure, unit–cell constant, and dielectric properties. A two-phase combination region was identified over the entire compositional range. The effect of sintering conditions was analyzed for various properties. Both permittivity (*ε_r_*) and Q factor (Q*_f_*) were sensitive to sintering temperatures and holding times, and the optimum performance was found at 1350 °C withholding time of 4 h. The temperature coefficient of resonant frequency (*τ_f_*) in a range from −45.2 to −52 (ppm/°C) and unit–cell constant were not sensitive to both the sintering temperature and holding time. An optimized Q factor of 192,000 (GHz) related with a permittivity (*ε_r_*) of 17.35 and a temperature coefficient (*τ_f_*) of −47 (ppm/°C) was realized for the specimen sintered at 1350 °C withholding time of 4 h. For applications of 5G communication device (filter, antennas, etc.), Mg_0.95_Ni_0.05_TiO_3_ is considered to be a suitable candidate for substrate materials.

## 1. Introduction

Due to the speedy evolution of communication technology, today’s 5G, and even the next generation of communication systems, component performance improvement and size reduction are currently the first two goals. Therefore, to be able to effectively reduce the size and improve the performance of components, quite a few laboratories place their attention on electronic ceramics. In the field of microwave (MW) dielectrics applications [1,2,3,4,5,6,7,8], electronic ceramics are subject to the following conditions:

1. High permittivity (*ε_r_*): the *ε_r_* is inversely related to the wavelength in dielectrics (λ= λ0εr), so the component size can be effectively reduced.
2. High-quality factor (Q factor, Q*_f_*): Q factors are inversely related to the dielectric loss (Q = 1/tanδ), so high Q factors can improve frequency selectivity and stability in MW components.
3. A temperature coefficient (*τ_f_*) close to zero: A *τ_f_* value close to zero ensures that component performance is not affected by external temperatures.

Magnesium titanium (MgTiO_3_) is a member of the ilmenite group which crystallizes in the trigonal system [9,10,11,12,13,14,15,16]. MgTiO_3_-based ceramics have been widely applied to dielectrics in resonators, filters, and antennas for communication, radar, and global positioning systems operated at microwave frequencies. They have a fairly low dielectric loss (4.5 × 10^−5^) and a minus temperature coefficient (*τ_f_* = −50 ppm/°C), when blending with CaTiO_3_, which has a perovskite structure and extensive plus temperature coefficient (*τ_f_*) ~ +800 ppm/°C. As blending ratio with 95:5, the combination was manifested a *τ_f_* value ~ 0 ppm/°C, a permittivity (*ε_r_*) ~ 21, and loss tan = 1.25 × 10^−4^ at 7 GHz [9,17,18,19]. However, because the sintering temperature (S.T.) of the phase combination is too high (up to 1450 °C), quite a lot of research has been done to effectively reduce its S.T. For example, improve the conditions of the process or add different sintering promoters. The dielectric performances of the combination can be further ameliorated by blending sintering addition such as Cr, La, or B [17,18,19].

The relevant research has pointed out that replacing magnesium with the 2 valence elements of 0.05 moles (such as Co^2^^+^, Zn^2^^+^), can get optimum characteristics [10,13,20]. With the partial replacement of Mg by Ni, (Mg_0.95_Ni_0.05_)TiO_3_ (MNT) ceramics with an ilmenite-type structure has been reported to possess excellent dielectric properties with a dielectric constant (*ε_r_*) of 17.2, a quality factor (Q × *f* value) of 180,000 (GHz), and a temperature coefficient of resonant frequency (*τ_f_*) ~−45 ppm/°C) [10]. In the past, many researches manifested that MNT blended with positive temperature coefficient material (such as CaTiO_3_, SrTiO_3_, Ca_0.8_Sr_0.2_TiO_3_, etc.) can be effectively reached close to zero temperature coefficient [20,21,22]. However, no studies have explored the characteristics of the pure MNT phase in depth until now. Therefore, we only know about the mixed-phase characteristics of MNT with blending positive *τ_f_* materials. In this article, we try to supersede magnesium (Mg^2+^: 0.072 nm) with trace amounts of nickel (Ni^2+^: 0.069 nm). Magnesium (Mg^2+^) superseded by nickel (Ni^2+^) of 0.05 mole can enhance the densification of microstructure and microwave performances in the phase of MNT. The combination of MNT phase was combined by a solid-state method. The relevant microwave dielectric performances were analyzed according to the densification, X-ray Diffraction (XRD) analysis, unit–cell constant, and microstructure of the phase ((Scanning Electron Microscope (SEM) and Energy Dispersive Spectroscopy (EDS)) The connection between sintering conditions and microwave performances is also further discussed in the pure MNT phase.

## 2. Results and Discussion

**Figure 1:**
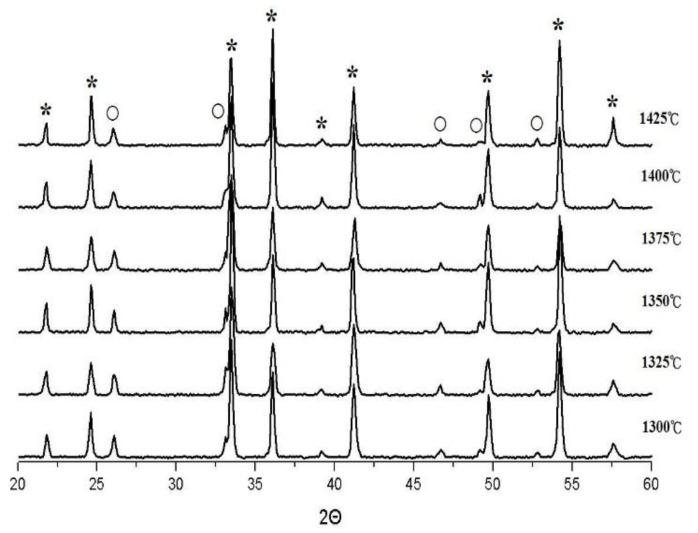
XRD analysis of MNT ceramics sintered at varied temperature holding times of 4 h. (*: MNT, ◯: MNT2).

**Figure 2:**
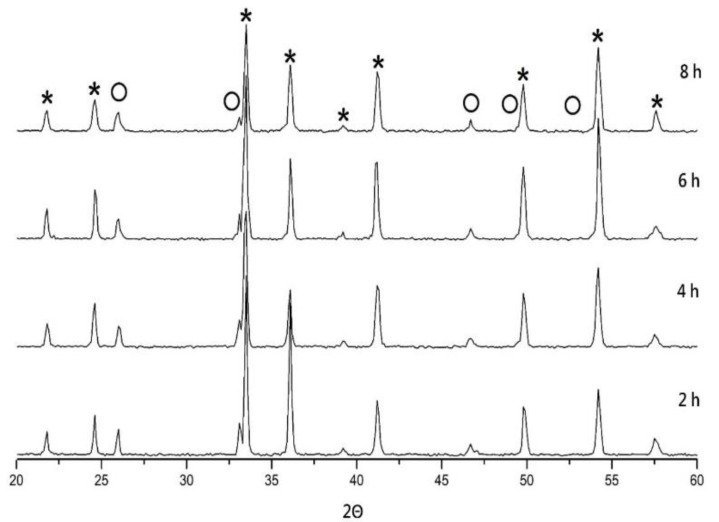
XRD analysis of MNT ceramics sintered at 1350 °C with varied holding time. (*: MNT, ◯: MNT2).

XRD analysis of MNT phase combination at varied S.T. (1300 °C–1425 °C) and holding time (2–8 h) are shown in Figure 1 and Figure 2, individually.

The MNT phase has an ilmenite-type structure, which is duplicated as magnesium titanium in the trigonal system (ICDD #06-0494). In this combination, MNT can be designated as the primary phase and a small fraction of the secondary phase Mg_0.95_Ni_0.05_Ti_2_O_5_ (MNT2 hereafter) existence. The MNT2 was duplicated as MgTi_2_O_5_ (JCPDS File No. 82-1125), which usually appears at MgO and TiO_2_ respond in a 1:2 mole ratio and is difficult to exhaustively remove in traditional solid-state reactions [23,24]. In this study, MNT2 was also designated as a secondary phase with permittivity (*ε_r_*)~13.1, Q factor ~30,000 GHz, and *τ_f_* ~−43 ppm/°C [25]. The microwave performance of the MNT phase may be diminished by the formation of the secondary phase. As expected, when the temperature rises, the ratio of the MNT2 phase rises because its growth conditions require a higher temperature of 1450 °C [25]. The relative percentage of MNT2 intensity raised from 10.4% to 13.2% as the S.T. raised from 1300 °C to 1425 °C. In contrast, this is also revealed at lower temperatures (<1450 °C), the proportion of the secondary phase will be less. Figure 2 shows that the same crystallization analysis results were held at 1350 °C for 2 to 8 h as mentioned above, with no significant change in both angle and intensity.

To further understand the structure and combination of the MNT phase, we have also calculated that the unit–cell constant of the MNT phase sintered at 1350 °C for a varied holding time as shown in Table 1. Compared with MgTiO_3_ (ICDD-PDF #00-006-0494), the unit–cell constant of the MNT phase tended to decrease. The results showed that when 0.05 moles of nickel (Ni^2+^) superseded magnesium (Mg^2+^), a solid solution of the MNT phase could be formed. The change of MNT phase unit–cell constant was mainly since the ion radius of nickel (Ni^2+^: 0.069 nm) is relatively smaller than that of magnesium (Mg ^2+^: 0.072 nm), which would lead to local changes in the unit–cell constant of MNT phase in a-site. This result indicates that when 0.05 mole nickel (Ni^2+^) supersedes magnesium (Mg^2+^) to form MNT phase, the unit–cell constant is reduced from (a = 0.5054, and c = 1.3898 nm) in MgTiO_3_ to (a = 0.5046, and c = 1.3905 nm).

**Figure 3:**
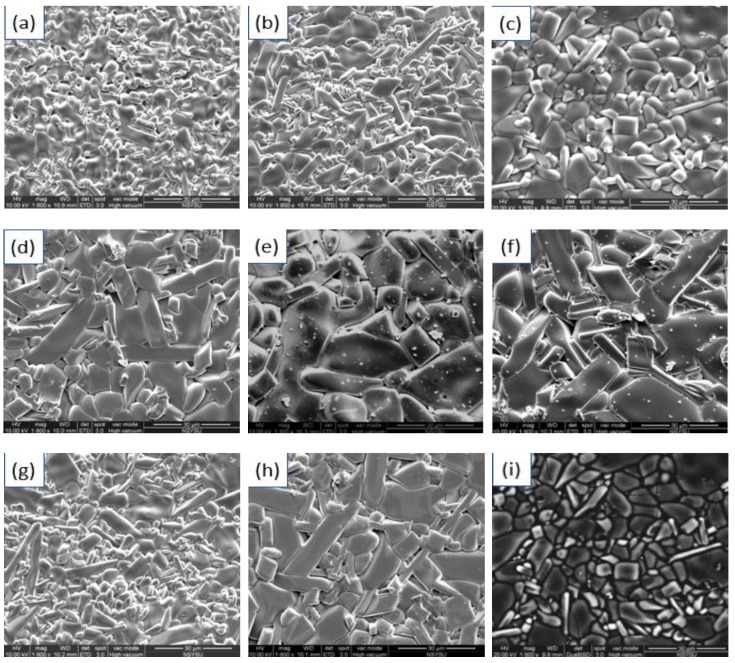
SEM photographs of MNT ceramics sintered at varied temperature holding times of 2–6 h and the BEI of MNT ceramics sintered at 1350 °C holding of 4 h: (**a**) 1300 °C; (**b**) 1325 °C; (**c**) 1350 °C; (**d**) 1375 °C; (**e**) 1400 °C; (**f**) 1425 °C; (**g**) 1350 °C/2 h; (**h**) 1300 °C/6 h; (**i**) BEI-1350 °C/4 h.

**Figure 4:**
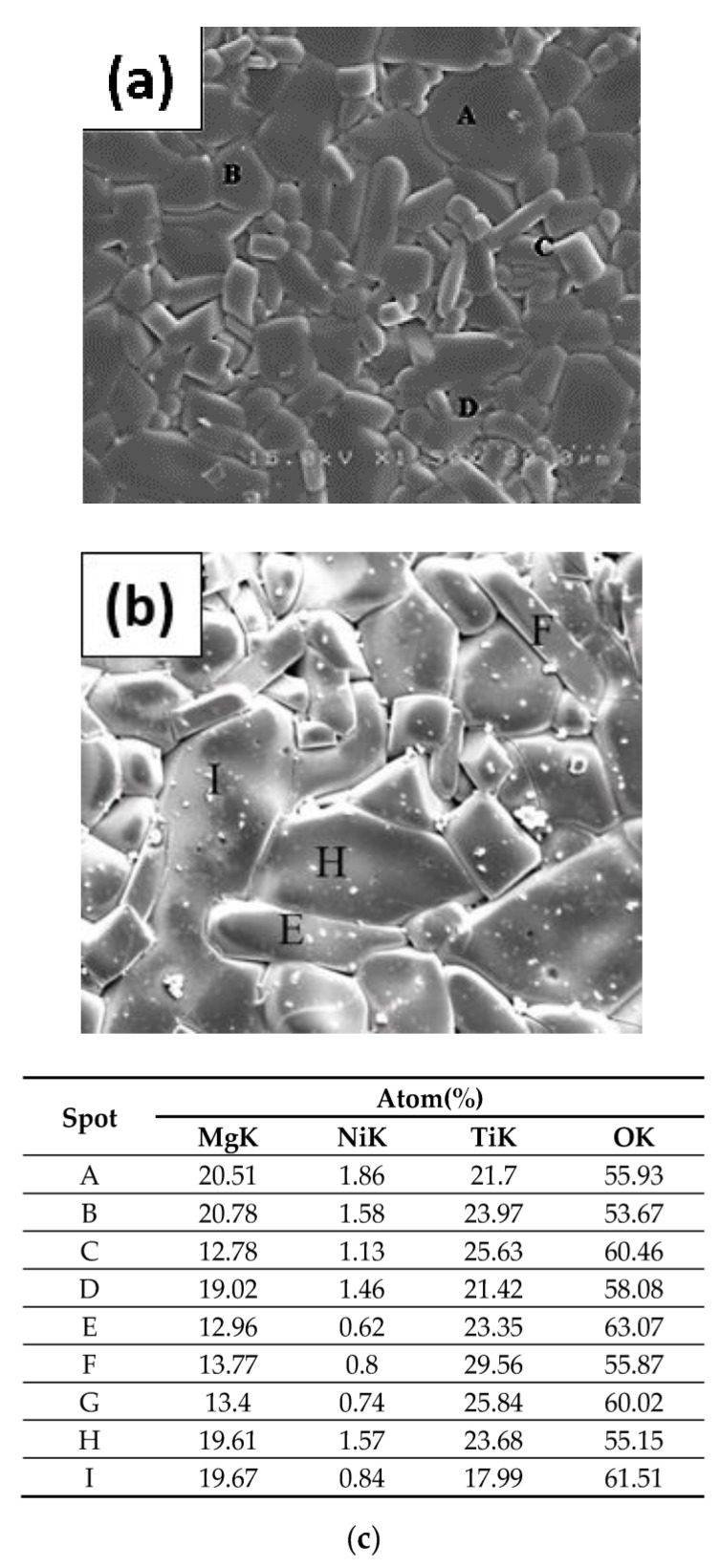
The EDS photographs of (**a**) MNT at 1350 °C (**b**) MNT2 at 1400 °C and (**c**) analysis of correspondent markings A–I.

To observe the growth of grains in crystalline structures, the surface microstructure photographs of MNT combinations at varied sintering conditions are shown in Figure 3a–f. It can be seen from the figure that at 1300 °C, the grains had not grown and the overall structure had not been compacted. The dimension of the grains rose with the rising of S.T., and it was more consistent to reach a highly uniform structure at 1350 °C. However, excessive S.T. (1400 °C) can lead to excessive grain expansion, which in turn can lead to an uneven arrangement in the structure and adversely affect the overall characteristics. Stick-like grain growth was enhanced as the S.T. higher than 1350 °C. This type of grain can be considered as MNT2 phase, the formation condition is that when the ratio of magnesium to titanium is 1:2, can be seen by the EDS analysis of Figure 4 (Spot C). MNT2 phases existed at higher temperatures as discussed earlier [26]. The EDS analysis of the specimen sintering at 1400 °C is also shown in Figure 4. It can be seen that the proportion of stick-like grain increases significantly at this S.T. Moreover, the microstructure of samples sintered at 1350 °C holding 2–6 h shown in Figure 3c,g,h sequentially revealed grains undergrown to overgrown process and well-grown grains was obtained at 1350 °C holding 4 h. As expected, MNT combinations were exhibited as primary phases associated with the apparent second phase MNT2 in the specimens. This was verified in the backscattered electronic (BEI) image shown in Figure 3h and EDS analysis in Figure 4.

**Figure 5:**
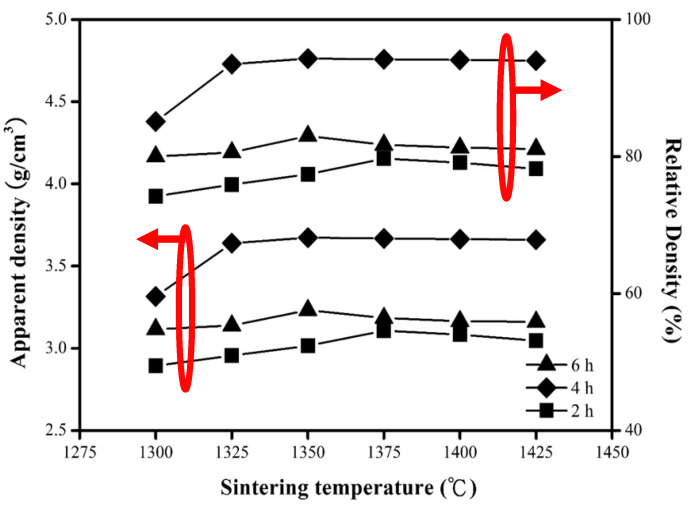
Dependence of apparent density and relative density on S.T. of the MNT ceramics for varied holding time. The left and right circles with arrows indicating apparent and relative density, respectively.

Figure 5 shows the measured and relative densities of the MNT combinations sintered at varied temperatures holding 2–6 h. The optimum measured and relative densities of the MNT ceramics were 3.67 g/cm^3^ and 94.3% inspected sintering at 1350 °C holding 4 h. The density of holding 4 h samples can be seen in the figure, reaching an optimal value as the S.T. rising to 1350 °C, and then showing a downward trend as the temperature still goes up. The rise in density is mainly due to the expansion of grains, while the downward trend is due to the uneven structure caused by excessive grain growth as shown in Figure 3. On the other hand, a variation in the sintering holding time (2–4 h) would also expand the grains, resulting in an increment in the density as shown in Figure 3c,f. However, the density was also diminished with the longer holding time (6 h) caused by excessive grain growth shown in Figure 3g. The measured density and its proportional TD rose from 3.31 g/cm^3^ (85.1% TD) to maximum values of 3.67 g/cm^3^ (94.3%TD) as the S.T. rose from 1300 °C to 1350 °C for the MNT combinations with sintering holding 4 h.

**Figure 6:**
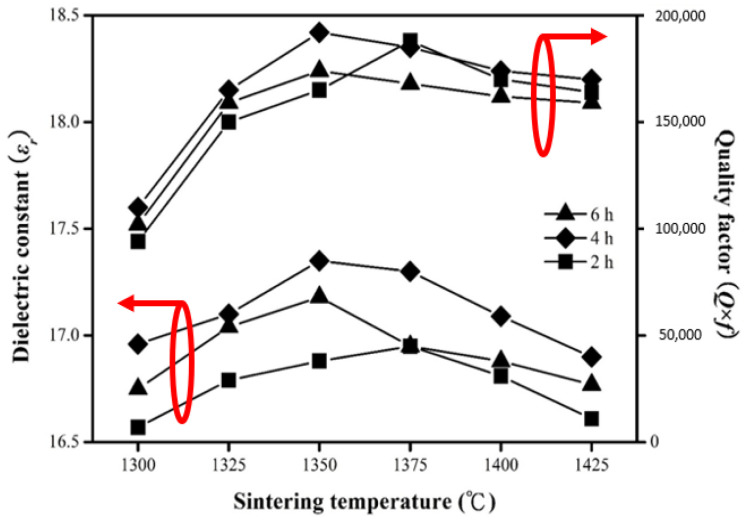
The dielectric constant and quality factor of the MNT ceramics as a function of the S.T. for varied holding time. The left and right circles with arrows Dielectric constant and Quality factor, respectively.

Figure 6 shows the results of permittivity (*ε_r_*) and Q factors (Q*_f_*) under different sintering conditions. The relationship between permittivity (*ε_r_*) and the S.T. has an equivalent orientation as those among ionic polarization, relative density, and S.T. since higher density expresses lower porosity. When the S.T. went up, the permittivity (*ε_r_*) rose slightly, and it can be found in the relevant literature that the permittivity (*ε_r_*) in dielectric materials is mainly dominated by ion polarization [27]. As mentioned above, when nickel (Ni^2+^) was superseded by magnesium (Mg^2+^), the permittivity (*ε_r_*) of MNT combinations descended. This may be because the replacement of nickel (Ni^2+^) causes some compression in the unit–cell structure, which in turn deviates ionic polarization [28]. Therefore, the permittivity (*ε_r_*) of MNT combinations is mainly determined by the ionic polarization. The relationship between the permittivity (*ε_r_*) and S.T shows an identical tendency as that among density and S.T. because higher density results in lower porosity as shown in Figure 3. However, descended of permittivity (*ε_r_*) was observed from 1375 °C holding 2 h. The rise in permittivity (*ε_r_*) could be demonstrated owing to higher densities. Thus, rising S.T. does needless upshot in a higher permittivity (*ε_r_*). The permittivity (*ε_r_*) of the well-sintered MNT combinations ranged from 16.9 to 17.35 at 1300–1425 °C holding 4 h. An optimum permittivity (*ε_r_*) of 17.35 was obtained for the MNT combinations sintered at 1350 °C holding 4 h.

The Q factor enhance with temperature rose to 1350 °C and then descended. An optimum Q factor of 192,000 (GHz) was acquired for MNT combinations at 1350 °C holding 4 h. The decline of the Q factors is mainly attributed to uneven structure due to excessive grain expansion at higher S.T., as shown in Figure 3. In general, the main causes of dielectric loss are the vibration mode of the unit–cell, pores, secondary phase, impurities, and structure defects [28]. As can be known from other relevant dielectric material performances, density is often one of the important factors in establishing dielectric-loss. On the other hand, the Q factors of MNT combinations would descend when the holding time surpassed 4 h. This descend was signified that excessive grain growth also occurred at a longer holding time.

**Figure 7:**
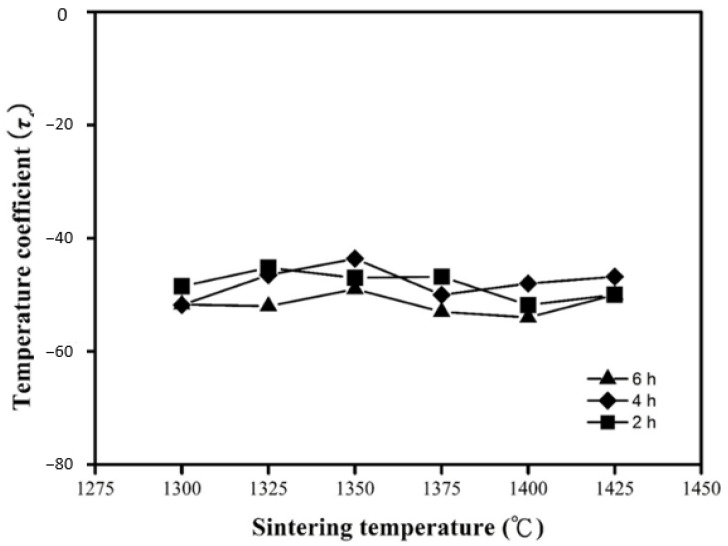
Dependence of *τ_f_* value on sintering temperature of MNT ceramics for varied holding times.

Figure 7 shows the temperature coefficients of resonant frequency (*τ_f_*) of the MNT combinations with varied sintering conditions. The *τ_f_* values were affiliated to the combination and the secondary phase of synthesized material in widespread. However, it seems to be closely related to the density of the proposed materials and sintering conditions. When the composition remained identical and no other secondary phases were observed, no remarkable variation in the *τ_f_* value can be seen as anticipated. The measured *τ_f_* values ranged from −45.2 to −51 ppm/°C as the specimen sintered at 1300 °C–1425 °C holding 4 h. At 1350 °C and holding 4 h, a *τ_f_* value of −47 ppm/°C was obtained for the MNT combination.

## 3. Experimental Procedure

The specimen of MNT ceramics was merged with high-purity chemical powders. The stoichiometric percentages of raw oxide powders were weighted and ball-grinded in alcohol using zirconia balls as a grinding medium. Afterward, the blend solution was parched at 90 °C and pre-sintered at 1100 °C holding 4 h. The pre-sintered powder was re-grinded in alcohol solution using zirconia balls and after parching, the polyvinyl alcohol was dropped into the powders as a binder and then crushed into a fine powder through a sieve. The gained powder was pressed into ingots under 100 MPa with 1 cm in diameter and 0.5 cm in thickness. The binder in these ingots was evaporated at 650 °C holding 2 h and then sintered at 1300–1425 °C for varied holding times as the heating rate of 10 °C/min.

The densities of ingots were measured and compute by using the Archimedes method. The permittivity and Q factor were measured using the Hakki–Coleman dielectric resonator methodology [29], as improved by Courtney [30]. The measurement system was connected to the Anritsu network analyzer with model MS46122B. The *τ_f_* value was measured with an identical setup, but in the thermostat extent from 20 °C to 80 °C. The following formula was utilized to obtain the *τ_f_* value (ppm/°C):

τf= f2−f1f1(T2−T1)

where *f*_1_ and *f*_2_ represented the resonance frequencies at *T*_1_ and *T*_2_, respectively.

## 4. Conclusions

In this paper, the sintering conditions in Mg_0.95_Ni_0.05_TiO_3_ ceramics and the influences on XRD analysis, microstructure, unit–cell constants, and microwave performance are systematically explored. The replacement of magnesium (Mg^2+^) with nickel (Ni^2+^) from 0.05 moles can form a Mg_0.95_Ni_0.05_TiO_3_ solid solution, which can significantly reduce dielectric-loss. In particular, the pure Mg_0.95_Ni_0.05_TiO_3_ combination possessed a high Q*f*~192,000 (GHz), which represents an approximately 21.9% reduction in the dielectric loss compared with that of the pure MgTiO_3_ and retained comparable *ε_r_* (~17.35) and *τ_f_* value (~−47 ppm/°C). A two-phase combination region was identified over the entire compositional range, and the existence of the Mg_0.95_Ni_0.05_Ti_2_O_5_ phase might diminish the dielectric performances of the example. The proposed dielectric combination, which has very low dielectric loss with appropriate permittivity and temperature characteristics, makes it a very promising candidate for practical applications in microwave and the communications systems of the new generation.

## Figures and Tables

**Table 1:** The unit–cell constant of the MNT phase with varied holding times.

S.T.	Holding Time	a (nm)	c (nm)
1325 °C	2 h	0.5024 ± 0.00197	1.38518 ± 0.00398
	4 h	0.50395 ± 0.00100	1.38357 ± 0.00200
	6 h	0.50429 ± 0.00125	1.38971 ± 0.00254
1350 °C	2 h	0.50439 ± 0.00135	1.38850 ± 0.00273
	4 h	0.50459 ± 0.00126	1.39054 ± 0.00256
	6 h	0.50611 ± 0.00070	1.38949 ± 0.00141
1375 °C	2 h	0.50288 ± 0.00163	1.39250 ± 0.00335
	4 h	0.50386 ± 0.00080	1.38478 ± 0.00161
	6 h	0.50401 ± 0.00116	1.38811 ± 0.00235
